# Sepsis-Exacerbated Brain Dysfunction After Intracerebral Hemorrhage

**DOI:** 10.3389/fncel.2021.819182

**Published:** 2022-01-21

**Authors:** Jie Lin, Binbin Tan, Yuhong Li, Hua Feng, Yujie Chen

**Affiliations:** ^1^State Key Laboratory of Trauma, Burn and Combined Injury, Department of Neurosurgery, Southwest Hospital, Third Military Medical University, Army Medical University, Chongqing, China; ^2^Chongqing Clinical Research Center for Neurosurgery, Southwest Hospital, Third Military Medical University, Army Medical University, Chongqing, China; ^3^Chongqing Key Laboratory of Precision Neuromedicine and Neuroregenaration, Southwest Hospital, Third Military Medical University, Army Medical University, Chongqing, China

**Keywords:** intracerebral hemorrhage, sepsis, inflammatory, neuronal death, brain dysfunction

## Abstract

Sepsis susceptibility is significantly increased in patients with intracerebral hemorrhage (ICH), owing to immunosuppression and intestinal microbiota dysbiosis. To date, ICH with sepsis occurrence is still difficult for clinicians to deal with, and the mortality, as well as long-term cognitive disability, is still increasing. Actually, intracerebral hemorrhage and sepsis are mutually exacerbated *via* similar pathophysiological mechanisms, mainly consisting of systemic inflammation and circulatory dysfunction. The main consequence of these two processes is neural dysfunction and multiple organ damages, notably, *via* oxidative stress and neurotoxic mediation under the mediation of central nervous system activation and blood-brain barrier disruption. Besides, the comorbidity-induced multiple organ damages will produce numerous damage-associated molecular patterns and consequently exacerbate the severity of the disease. At present, the prospective views are about operating artificial restriction for the peripheral immune system and achieving cross-tolerance among organs *via* altering immune cell composition to reduce inflammatory damage.

## Introduction

Intracerebral hemorrhage (ICH) is frequently accompanied by infection ranging from 11 to 31% and long-term functional impairment (Ali et al., 2009; Lord et al., 2014). The majority of infectious patients will rapidly deteriorate and finally develop sepsis, due to systemic metabolic disorders and stress caused by excessive release of inflammatory factors and immunosuppression after ICH (Berger et al., 2014; Cheng et al., 2018). Clinically, sepsis complicated by the ICH is common but tricky in the neurosurgical intensive care unit and kills as many as a half (Goncalves et al., 2019). Our retrospective cohort study has shown that approximately 28% of patients with ICH would accompany sepsis, and sepsis is the leading cause of poor outcomes. Furthermore, approximately 80% of survivors will face severe sequelae of various organ damages, especially in the brain (Adam et al., 2013). Actually, there are many synergies in the pathophysiological mechanism of both ICH and sepsis. For example, systemic inflammation after either ICH or sepsis emerges as a crucial trigger and mediator in the progression of secondary insult to the brain (Adam et al., 2013; Fu et al., 2015). In addition, subsequent circulatory dysfunction can be observed in both situations, leading to worse damage progress (Taccone et al., 2010; Kopeikina et al., 2020).

To date, ICH with sepsis is still difficult for clinicians to deal with, and the mortality and long-term cognitive disability are still increasing. Thus, understanding the relevant pathophysiology seems to be imminent and will be beneficial for the exploration of specific therapies. In this review, we focus on the crosstalk between ICH and sepsis and attempt to identify the mechanism of cerebral dysfunction, aiming to provide a unique and systematic insight into the interaction of the two diseases and guide indications for clinical treatment.

## Pathophysiology

**FIGURE 1:**
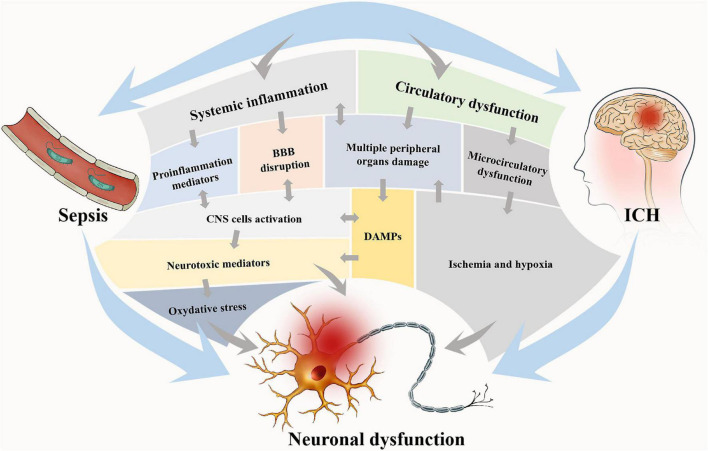
Two main pathophysiological processes are involved in brain dysfunction in ICH with sepsis. These two processes are interdependent, on one hand, and mutually independent, on the other hand. Circulatory dysfunction will be followed by microcirculatory dysfunction and finally result in ischemia and hypoxia of tissues and multiple peripheral organ damages. In systemic inflammation, proinflammatory mediators will be released, and the DAMPs from the periphery will be allowed into the brain due to BBB disruption, which consequently activates CNS cells. The main consequence of these two processes is neural dysfunction, notably *via* oxidative stress and neurotoxic mediation. Neural dysfunction widely exists in the brain and accounts for the brain dysfunction *via* the alteration of neurotransmission. DAMPs, damage-associated molecular patterns; BBB, blood-brain barrier; CNS, central nervous system.

Intracerebral hemorrhage and sepsis are mutually exacerbated *via* several pathophysiological mechanisms mainly consisting of systemic inflammation and circulatory dysfunction (see Figure 1).

### Systemic Inflammation

On the onset of ICH, primary damage caused by disruption of normal anatomy occurred pathologically in a limited area and time window (Sun et al., 2016). Subsequently, the release of blood components [red blood cells (RBCs), thrombin (Babu et al., 2012), hemoglobin, and hemin (Robinson et al., 2009; Babu et al., 2012)], coagulation factors, complement components, and immunoglobulins activate multiple cerebral cells such as endothelial cells, microglia, and astrocyte, which is followed by proinflammatory cytokines release (Wagner et al., 2002; Nakamura et al., 2005; Aronowski and Zhao, 2011). As a result, the expression of Toll-like receptors (TLRs) and adhesion-related molecules (ARMs) is upregulated (Kodali et al., 2021). Furthermore, TLRs, as a group of class I transmembrane proteins, are critical to identifying the pathogen-associated molecular patterns (PAMPs) from bacteria (Zhu and Mohan, 2010; Fitzgerald and Kagan, 2020) and damage-associated molecular patterns (DAMPs) from systemic inflammatory injury (Kong and Le, 2011). Owing to the above contributors, the cerebral cells will transform into a “hyper-alert state” and become highly sensitive to exogenous substances and active signals. Thus, it is sepsis insult in patients with ICH, which is similar to adding fuel to the fire. The peripheral immune cells are selected and activated by invasive bacteria or toxins in circulation, secreting a series of inflammatory cytokines to induce the systemic inflammatory responses, which further amplify cerebral cell signal cascades (Singer et al., 2018) and bring catastrophic damage to the central nervous system (CNS).

### Circulatory Dysfunction

In parallel, circulatory dysfunction can be observed after ICH and sepsis owing to pathological hypoperfusion and coagulation system disorders (Zheng and Wong, 2017; Font et al., 2020). During this process, inflammatory mediators first trigger the endothelial cells to express typical ARMs such as vascular cell adhesion molecule-1 (VCAM-1), intracellular cell adhesion molecule-1 (ICAM-1), endothelin-1, and platelet/endothelial cell adhesion molecule (Machado-Pereira et al., 2017). Especially, endothelin-1 is associated with continuous cerebral vasospasm resulting in local brain ischemia and hypoxia (Zheng and Wong, 2017). Activation of coagulative factors and formation of white/red blood cell plugs are also participating in the ischemic process. Excessive thrombin activation and platelet consumption are implicated with disseminated intravascular coagulation in the late stage (Goyette et al., 2004). Virtually, in the setting of systemic inflammation, the cerebral blood vessels are initially affected by CNS and that mediates further cytokine-dependent signals (Wong et al., 1996; Laflamme and Rivest, 1999). It has been confirmed that macro- and microcirculatory failure occurred rapidly and that is attributed to neurovascular coupling disorder (Rosengarten et al., 2009). Subsequently, extensive cells, especially brain cells, are damaged by compromised supplements of oxygen, nutrients, and metabolites (Sharshar et al., 2004). In turn, these damage signals can feedback to the central and peripheral cells and further augment systemic inflammation, which predisposes to a vicious circle of CNS dysfunction.

## Sepsis Susceptibility Increased by Intracerebral Hemorrhage

### Immunosuppression

The immune system will undergo a profound attenuation process in the setting of severe CNS injury (including traumatic brain injury, stroke, and spinal cord injury) (Fu et al., 2015). A meta-analysis consisting of 137,817 patients has identified the correlation between the high rates of systemic infections and stroke, and reported that approximately 30% of patients with stroke were along with infection including pneumonia or urinary tract infection (Westendorp et al., 2011). Temporary lymphopenia and splenic shrunk can be observed in both humans and animals at the early stage of stroke, *via* activation of the sympathetic, parasympathetic (cholinergic anti-inflammatory), and hypothalamus-pituitary-adrenal (HPA) axis pathways (Ajmo et al., 2009; Sahota et al., 2013). Thereby, the levels of noradrenaline, acetylcholine, and glucocorticoids in circulation are abruptly elevated by the promotion of these active neuroendocrine pathways, which are responsible for apoptosis and atrophy of lymphoid organs (Wong et al., 2011; Mracsko et al., 2014). This downregulation of immune cell generation and function that originates from the injured brain is aiming to avoid autoimmunity against brain antigens from the death or impaired cells (Fu et al., 2015), whereas it causes systemic immunosuppression and makes the body vulnerable to infections simultaneously (Hug et al., 2009).

### Intestinal Microbiota Dysbiosis

The gut vascular barrier (GVB) mainly comprises three defense lines including the biological barrier set up by gut microbiota (Assimakopoulos et al., 2007) and keeps intestinal homeostasis. Intestinal flora displays important metabolic, immunologic, and gut protective functions modulated by the so-called “gut-brain axis” (Haak et al., 2018). Numerous models have shown that microbiota have the potentials to augment the proinflammatory effect of immune cells and even conduct the influx of immune effector cells into distant organs, probably mediated by microbe-associated molecular patterns including lipopolysaccharide (LPS), peptidoglycan, flagellin, and microbiota-derived metabolites (Magnotti et al., 1998; De-Souza and Greene, 2005). Moreover, the microbiota can induce the secretion of antibacterial factors from the gut-epithelial cells and, consequently, augment humoral responses against invading pathogens (Kim et al., 2017). ICH occurrence disturbs GVB integrity and intestinal hemostasis and, ultimately, alters the microbiota composition (Patterson et al., 2019; Rice et al., 2019). A recent study also has confirmed the prominent reduced species diversity and microbiota overgrowth in the dysbiosis induced by ICH, which may reduce intestinal motility and increase gut permeability. While recolonizing, normal health microbiota therapy ameliorated neural deficits and inflammation after ICH (Yu et al., 2021). Thus, it provides an opportunity for the potential translocation of aerobic opportunistic pathogens whereby impaired GVB and, finally, results in the onset of gut-origin sepsis (Donskey, 2004; Haak et al., 2017; Huber-Lang et al., 2018).

## Sepsis-Associated Inflammatory Signals to Central Nervous System Enhanced on Intracerebral Hemorrhage

### Signal Pathways in Central Nervous System Response to Sepsis Threat: Neural, Humoral, and Blood-Brain Barrier Alteration

There are three pathways to capture sepsis signals by CNS (Ali et al., 2009). Nervous pathways, mainly initiated by PAMPs and inflammatory cytokines *via* the primary afferent (vagal and trigeminal) and sensorial (olfactory) nerves (Sonneville et al., 2013). At this time, visceral inflammation due to sepsis can be detected by the vagal nerve depending upon its terminal cytokine receptors (Tracey, 2009). In addition, the vagal nucleus transmits signals to the central autonomic system, the neuroendocrine centers, and the amygdala, leading to the alteration of behaviors and emotions (Adam et al., 2013; Lord et al., 2014). Humoral pathways, conducted by circulating inflammatory mediators through choroid plexus (CP) and circumventricular organs (CVOs) (Sonneville et al., 2013). CVOs are defined as the fenestrated regions lacking the intact blood-brain barrier (BBB) around the brain, and hence, molecules and peripheral cells can directly access the cerebral parenchyma through these regions (D’Mello and Swain, 2014). Similar to CVOs structure lacking BBB, CP is constituted of cuboidal epithelium cells and is responsible for secreting cerebrospinal fluid (Ghersi-Egea et al., 2018). Both of these structures express receptors of innate and adaptive immune systems, allowing them to detect central and peripheral inflammatory signals (Adam et al., 2013; Ghersi-Egea et al., 2018). Once relevant signals were captured, they will be amplified and transmitted to deeper areas implicated with controlling behavioral, neuroendocrine, and neurovegetative responses *via* above two structures (Adam et al., 2013; Berger et al., 2014). BBB alteration, enabling monocytes infiltration, and inflammatory molecules invading in the systemic inflammation (Meneses et al., 2019). Activated endotheliocytes express ARMs and release several inflammatory mediators such as cytokines, prostaglandins, and nitric oxide (Johnston and Webster, 2009). It is involved in the regulation of neurotransmission and neurosecretion (Johnston and Webster, 2009). Studies have reported that both pro-inflammatory cytokines [i.e., tumor necrosis factor (TNF)-α and interleukin (IL)-6 and IL-1β] and anti-inflammatory cytokines (i.e., IL-1Ra and IL-10) collectively participated in these systemic responses and formed inflammation homeostasis (Wong et al., 1997; Ilyin et al., 1998; Pintado et al., 2011). However, the abrupt presence of ICH or sepsis breaks the balance and causes an inflammatory signal cascade.

### Central Nervous System Innate Cells-Associated Responses: Endothelial Cell Activation, Immunocyte Activation, and Blood-Brain Barrier Alteration

Endothelial cell activation is a crucial step in the CNS responses to sepsis, which affects microcirculatory function and BBB integrity. Evidence confirmed that LPS could induce various ARM expressions on the endothelial cells such as CD40, E-selectin, VCAM-1, and ICAM-1 (Hess et al., 1996; Wong et al., 1997; Omari and Dorovini-Zis, 2003; Hofer et al., 2008). In addition, several receptors for IL-1, TNF-α, and TLR4 were also upregulated (Wong et al., 1997; Zhou et al., 2009). And these receptors contribute to the secretion of TNF-α, IL-6, and IL-1β, followed by the generation of endothelial/inducible nitric oxide synthase (Freyer et al., 1999; Handa et al., 2008) and type-2 cyclooxygenase (Matsumura et al., 1998). In virtue of these inflammatory mediators, microglia are mobilized to secrete several cytotoxic molecules (Meneses et al., 2019), and astrocytes are also triggered to produce chemokines (the C-C chemokine ligand 2, IL-6, chemokine C-X-C ligand10) *via* NF-κB pathways (Mayo et al., 2014). Furthermore, the expression of ARMs and TLR4 as well as the conduction of chemokines collectively choose infiltration of peripheral monocytes and their participation in neuroinflammation (Adam et al., 2013). Microcirculatory dysfunction has been widely observed in multiple sepsis models owing to the aggregation of circulating white cells and monocytes in the CNS capillaries, compromising supplements of oxygen, nutrients, and metabolites (Bohatschek et al., 2001; Hofer et al., 2008; Zhou et al., 2009; Taccone et al., 2010). The BBB alteration has been clearly confirmed whereby versatile methods, including blue Evans, fluorescent-labeled dextran clearance, labeled granulocytes, electron microscopy, and magnetic resonance imaging in sepsis models and also in patients (Papadopoulos et al., 1999; Esen et al., 2005, 2012; Sharshar et al., 2007; Handa et al., 2008; Bozza et al., 2010). Furthermore, a recent study has confirmed that BBB displayed a short-term closure at the early stage of inflammation and gradually opened up as the disease progressed (Carloni et al., 2021). Nonetheless, it eventually allows for the entry of neurotoxic molecules, particularly inflammatory mediators, consequently giving rise to brain cell death in systemic inflammation. Likewise, extensive studies have found that the neuroinflammation in the ICH was complicated with gliacyte activation and BBB alteration (Anrather and Iadecola, 2016; Wofford et al., 2019). Therefore, all above mentioned mechanisms indicate that the occurrence of ICH greatly increases the risk of sepsis.

## Neural Death on Intracerebral Hemorrhage With Sepsis

Intracerebral hemorrhage and sepsis can cause similar patterns of neural death and are discussed in the following text (Table 1; Castagna et al., 2016; Oberst, 2016; Lewerenz et al., 2018; Li et al., 2018; Takashima et al., 2019; Nagase et al., 2020). Reactive oxygen species (ROS), a kind of physiological defense molecule, can be maintained at a steady level *via* mitochondrial oxidative phosphorylation and antioxidant mechanisms (Zhou et al., 2020). However, the onset of ICH or sepsis overgenerates ROS and then causes mitochondrial damage, leading to the deterioration of iron metabolism (Zhou et al., 2020; Liu et al., 2021). Subsequently, hemin released from RBC lysis owing to the elevation of cytokines or bacterial toxins also accounts for excessive free iron in the extracellular matrix (Soares and Weiss, 2015; Ganz, 2016). Subsequently, extracellular iron bounding to the transferrin receptor is internalized by the cells under the drive of inflammatory cytokines (Ludwiczek et al., 2003). Excess iron in the cytoplasm significantly dampens enzyme activity and typically causes potent oxidization, resulting in various cell ferroptosis including neurocytes (Liu et al., 2021). Numerous reports have indicated that ferroptosis is invariably followed by necroptosis (Zhou et al., 2020), and NADPH might be the connectional mediator between the two patterns of cell death (Hou et al., 2019). In systemic inflammation, necroptosis can be initiated by several cytokines including, but not limited to, TNF (Oberst, 2016). Once ferroptosis or necroptosis happened, adjacent cells are likely predisposing to another kind of death pattern especially oxytosis (Zhou et al., 2020). Highly similar to the mechanism of ferroptosis, oxytosis occurrence is also related with the extensive ROS failed to be metabolized because of glutathione depletion (Landshamer et al., 2008; Grohm et al., 2010). Many studies even regarded oxytosis as a component of ferroptosis (Soares and Weiss, 2015; Zhou et al., 2020), and this needs further research to clarify. Apoptosis has been well studied by numerous researchers and mainly conducted by two pathways, namely extrinsic and intrinsic pathways. Among them, the extrinsic pathway is activated by cell surface receptors including TNF receptors (Hasegawa et al., 2011; Fricker et al., 2018; Zhao et al., 2018). Under the condition of systemic inflammation in ICH or sepsis, cerebral proinflammatory factors (e.g., TNF) are released in large quantities, and consequently, the Fas-associated death domain protein can be chosen to activate caspase-8 causing neural apoptosis (Micheau and Tschopp, 2003). Pyroptosis is one of the characteristic manners of cell death upon inflammation. In experimental models, it has been demonstrated to be induced by proinflammatory cytokines (i.e., IL-1β and IL-18) *via* the combination on the cell membrane between the lipid-selective N-terminal domain and phosphatidylinositol of the lipid plasma membrane (Shi et al., 2015; Ding et al., 2016; Feng et al., 2018). To sum up, inflammatory cytokines and metabolites are prioritized to induce neural death in the ICH with sepsis. Therefore, inhibition of neuroinflammation might be important for curbing brain function deterioration and warrant to be further explored.

**TABLE 1:** Neuronal death patterns of both ICH and sepsis.

Types	Activators	Characterization	References
Ferroptosis	Iron and extracellular glutamine	Plasma membrane integrity loss, organelles disruption and swelling, mitochondria shrunk, without DNA fragmentation	Li et al., 2018
Necroptosis	Inflammatory factors	Plasma membrane integrity loss, organelles disruption and swelling, without mitochondria shrunk, without DNA fragmentation	Oberst, 2016; Li et al., 2018
Apoptosis	Inflammatory factors	Chromatin condensation, nuclear shrinkage, and DNA fragmentation	Castagna et al., 2016; Li et al., 2018
Oxytosis	Glutamate; ROS	Mitochondrial fragmentation, without DNA fragmentation	Lewerenz et al., 2018; Takashima et al., 2019; Nagase et al., 2020
Pyroptosis	Inflammatory factors	Nuclear condensation, cell swelling, lipid membrane vacuole formation, lipid membrane ruptures, without DNA fragmentation	Li et al., 2018

## Multiple Organ Damage-Associated Molecular Patterns Release Exacerbates Central Nervous System Dysfunction

### Inflammatory Imbalance

Acute brain injury including ICH occurrence will initiate neuroinflammation and then spread inflammatory signals to the periphery, and monocyte infiltration might be a crucial mediator in this process. In an LPS-induced neuroinflammatory mouse model, the infiltrated neutrophils exhibited reverse trans-endothelial migration back to the bloodstream after interacting with microglia (Kim et al., 2020). Subsequently, these reverse-moving neutrophil-transported signals to several organs have been reported in numerous studies. For example, the upregulation of inflammatory cytokines (e.g., IL-8 and IL-10) were observed in the kidney used for organ donation, resulting in the reduction of allograft survival *via* increasing the number of trafficking inflammatory cells (i.e., FoxP3^+^ regulatory T cells) (Morariu et al., 2008; Kim et al., 2020). In addition, in the brain injury models, infiltrated monocytes and macrophages were demonstrated to produce several chemoattractants (i.e., leukotriene-B4) and other cytokines (e.g., IL-1β, IL-6, and TNF-α), causing the amplification of pulmonary inflammation (Kalsotra et al., 2007; Mrozek et al., 2015). In addition, other organ damages such as the spleen (Li et al., 2011), gastrointestinal tract (Fung et al., 2017), and liver (D’Mello and Swain, 2014) owing to neuroinflammation have been reported in previous studies. Meanwhile, in sepsis, the reactions of the host to invasive bacteria or toxins typically induce phagocytosis in macrophages and secrete a series of proinflammatory cytokines (Takeuchi and Akira, 2010). Besides, this so-called “cytokines storm” subsequently activates the innate immune system (D’Elia et al., 2013). Apparently, the activation of the innate immune system, which is modulated by pattern-recognition receptors, upregulates the expression of associated inflammatory genes *via* detecting PAMPs or DAMPs (Raymond et al., 2017). Thus, the simultaneous occurrence of ICH and sepsis can elicit the superposition of inflammatory effects and cause extensive organ damages. Obviously, the inflammatory imbalance represents the most important basis for brain dysfunction pathogenesis in the ICH with sepsis and occurs throughout the whole process of the ICH with sepsis.

### Ischemia Process

Ischemia processes, consisting of microcirculatory dysfunction and macrocirculatory dysfunction, can be observed in the ICH and sepsis development. Furthermore, microcirculatory dysfunction arising from endothelial cell activation has been well-demonstrated in various sepsis models (Taccone et al., 2010). Meanwhile, endothelial cell dysfunction also activates the coagulation system to participate in the ischemia process (Adam et al., 2013). Similar processes have been reported in acute cerebral injury patients with vasospasm. It is presented as persistent narrowing of cerebral arteries and is believed to be contributed by spasmogenic or neuroinflammatory factors (Kassell et al., 1985; Taccone et al., 2010), which indicates the role of inflammation in the ischemia process. In addition, severe inflammatory responses caused by sepsis will disturb neurovascular coupling, followed by a disorder of heart rate and blood pressure and deterioration of macrocirculation (Godin and Buchman, 1996; Jafari and Damani, 2020). It has been reported the autonomic controlling system of the heart and vessel is compromised in polymicrobial sepsis because of the degraded autonomic nervous system (Pancoto et al., 2008). Besides, the parasympathetic nervous system (e.g., vagal nerve) is regarded as one of the critical pathways connecting the center and periphery, suggesting that the autonomic nervous system functions and inflammation may be interdependent (Huston and Tracey, 2011). The damage to the susceptible regions of the CNS, whether chemical or mechanical nature, can augment the sympathetic nerve or HPA axis, further causing the dysregulation of catecholamine and dopamine secretion (Lattanzi et al., 2018). The amount of catecholamine released into the bloodstream will activate α-receptors on the cell surface to produce vasoconstriction for abdominal viscera, leading to consequent hypoperfusion and ischemic injury (Lattanzi et al., 2018). In conclusion, the ischemia process in the ICH accompanied with sepsis is under the co-modulation of inflammation and neuroendocrine changes.

### Damage-Associated Molecular Patterns Generation and Insult

Damage-associated molecular patterns are non-microbial molecules in the host nucleus or cytoplasm and consist of high mobility group box 1 (HMGB1), histones, and adenosine triphosphate (Sunden-Cullberg et al., 2005; Ekaney et al., 2014; Zhou et al., 2015). Once released to the extracellular matrix from injury cells, they will act as the effective activators of the immune system and perpetuate non-infectious inflammatory responses to cause systemic inflammation and cellular injury, even death (Matzinger, 1994; Seong and Matzinger, 2004; Rubartelli and Lotze, 2007). In addition, the exact mechanisms may be implicated with the proinflammatory cytokines and chemokines secreted from active macrophages/microglia, which facilitates excessive neutrophil activation and infiltration into the tissues (Denning et al., 2019). Activated neutrophils can generate several kinds of toxic mediators including ROS and inducible nitric oxide synthase (iNOS) to cause oxidative stress and cellular injury (Gentile and Moldawer, 2013; Schaefer, 2014; Brinkmann, 2018). TLRs are considered as the pivotal signal receptors for DAMPs. Rodriguez-Yanez et al. (2012) have reported in a recent clinical study that the upregulation of TLR2 and TLR4 in the peripheral monocytes is closely related to the undesired prognosis in patients with ICH. In addition, the improved neurological function after ICH onset was demonstrated in the TLR4-knockout rodent (Sansing et al., 2011; Lin et al., 2012). HMGB1, as one of the DAMPs extensively discussed, can enable microglia to increase the NF-κB activity and the transcription of cyclooxygenase-2, TNF-α, and IL-1β (Yang et al., 2011). In turn, the TNF-α can feedback on microglia to facilitate the release of HMGB1 (Wang et al., 2015). Evidence indicated that HMGB1 may be contributive to the poor outcomes after CNS injury and the serum levels, which was associated with the disease severity (Nakahara et al., 2009; Zhou et al., 2010).

In summary, under the effect of inflammatory imbalance and ischemia, injury and/or death cells in multiple organs release DAMPs into circulation and amplify systemic inflammation, accompanying oxidative stress and cytotoxic mediator production, further causing more damage and brain dysfunction worsening.

## Perspective and Conclusion

Increasing evidence has indicated that there is an inextricable inflammatory association between the center and periphery. CNS injury will spread the danger signals to the periphery, and *vice versa*. Under the mediation of systemic inflammation and circulatory dysfunction, the pathological changes can be observed in multiple organs of the patients with ICH or sepsis. Recent advances in the multi-omics analysis could provide vivid evidence regarding the cascade of biofluids from the injured brain, namely, cerebrospinal fluid and circulation blood, and urine and saliva. In contrast, clinical therapies for patients with ICH with sepsis frequently focus on a single organ or system lacking holistic ideas, causing dissatisfied outcomes. For the ICH process, sepsis presence displays an aggravation to peripheral inflammation imbalance that should not be ignored. Although there are many methods attempting to modulate the peripheral immune system, such as antibiotic prophylaxis and probiotic therapy, and have shown limited achievement (Kim and Cho, 2021), the prospective views considered that we should operate artificial restriction for the peripheral immune system and achieve the cross-tolerance among organs *via* altering immune cell composition. Based on that, stem cell therapy was extensively used in clinical trials for diverse diseases including hemorrhagic stroke, and exhibited many advantages. Therefore, further study for crosstalk between center and periphery might be beneficial for us to explore potential methods for improving brain dysfunction and prognosis in patients with ICH or sepsis or combination.

## Author Contributions

JL, BT, and YL drafted the manuscript, and prepared the figure and table. HF and YC proofread and revised the manuscript, then gave the final approval for this submission. All authors contributed to the article and approved the submitted version.

## Conflict of Interest

The authors declare that the research was conducted in the absence of any commercial or financial relationships that could be construed as a potential conflict of interest.

## Publisher’s Note

All claims expressed in this article are solely those of the authors and do not necessarily represent those of their affiliated organizations, or those of the publisher, the editors and the reviewers. Any product that may be evaluated in this article, or claim that may be made by its manufacturer, is not guaranteed or endorsed by the publisher.
